# Cancer-specific mortality in breast cancer patients with hypothyroidism: a UK population-based study

**DOI:** 10.1007/s10549-022-06674-5

**Published:** 2022-07-31

**Authors:** Lauren McVicker, Christopher R. Cardwell, Stuart A. McIntosh, Úna C. McMenamin

**Affiliations:** 1 Centre for Public Health, Queen’s University Belfast, Belfast, Northern Ireland, UK; 2 Patrick G Johnston Centre for Cancer Research, Queen’s University Belfast, Belfast, Northern Ireland, UK; 3 Breast Surgery Department, Belfast City Hospital, Belfast Health and Social Care Trust, Belfast, Northern Ireland, UK

**Keywords:** Breast cancer, Hypothyroidism, Cancer-specific mortality, All-cause mortality, Population-based study

## Abstract

**Purpose:**

Epidemiological studies have indicated a higher prevalence of hypothyroidism in breast cancer patients, possibly related to shared risk factors and breast cancer treatments. However, few studies have evaluated how hypothyroidism impacts survival outcomes in breast cancer patients. We aimed to determine the association between hypothyroidism and breast cancer-specific and all-cause mortality.

**Methods:**

We conducted a population-based study using the Scottish Cancer Registry to identify women diagnosed with breast cancer between 2010 and 2017. A matched comparison cohort of breast cancer-free women was also identified. Using hospital diagnoses and dispensed prescriptions for levothyroxine, we identified hypothyroidism diagnosed before and after breast cancer diagnosis and determined associations with breast cancer-specific and all-cause mortality. Cox proportional hazards regression was used to calculate hazard ratios (HR) and 95% confidence intervals (CI) adjusted for potential confounders.

**Results:**

A total of 33,500 breast cancer patients were identified, of which 3,802 had hypothyroidism before breast cancer diagnosis and 565 patients went on to develop hypothyroidism after. Breast cancer patients had higher rates of hypothyroidism compared with cancer-free controls (HR 1.14, 95% CI 1.01–1.30). Among breast cancer patients, we found no association between hypothyroidism (diagnosed before or after) and cancer-specific mortality (before: HR 0.99, 95% CI 0.88–1.12, after: HR 0.97, 95% CI 0.63–1.49). Similar associations were seen for all-cause mortality.

**Conclusion:**

In a large contemporary breast cancer cohort, there was little evidence that hypothyroidism, either at diagnosis or diagnosed after breast cancer, was associated with cancer-specific or all-cause mortality.

**Supplementary Information:**

The online version of this article contains supplementary material available 10.1007/s10549-022-06674-5.

## Introduction

Breast cancer is the most common cancer in females globally and over two million cases are diagnosed annually [1]. Although incidence rates remain high, breast cancer mortality rates in Europe are declining and the 10-year survival rate after diagnosis has increased to around 70% [2, 3]. The increased survival for breast cancer patients represents increased opportunity for the development of comorbidities that could detrimentally affect quality of life as well as prognosis [4, 5].

Hypothyroidism is characterised by insufficient levels of circulating thyroid hormones; specifically thyroxine (T4), along with an elevated thyroid-stimulating hormone (TSH) [6]. In Europe, the prevalence of hypothyroidism is between 0.2% and 5.3% in the general population, with the highest rates in older women [7]. Thyroid hormone replacement therapy in the form of levothyroxine is used to treat hypothyroidism and manage the imbalance of hormones to prevent fatigue, weight changes and cardiovascular issues [6].

Thyroid hormones are essential in normal cellular growth and metabolism [8, 9], but they have also been implicated in cancer cell proliferation and disruption of normal signalling pathways [10–13]. In breast cancer specifically, preclinical models have shown that T4 (natural or synthetic from levothyroxine [14]) stimulates breast cancer cell proliferation and supports tumour cell survival in vitro, which was more marked for oestrogen-dependent breast cancer cells [12, 15], suggesting a role for oestrogen receptors (ERs) [14]. In support, evidence from in vivo models has suggested that induced hypothyroidism (and therefore lower T4) reduces mammary tumour progression and promotes apoptosis [16, 17].

Some, but not all [18], epidemiological studies have reported a higher rate of hypothyroidism in breast cancer patients in comparison to the general population [19–24], possibly as a result of shared risk factors such as older age and female sex [25, 26] and cancer treatments such as radiotherapy to the lymph nodes which has been posited to damage thyroid cells directly [27, 28], and in combination with systemic therapies [23, 29]. Higher circulating levels of T4 have been suggested to be associated with improved breast cancer survival [30] but no such associations have been observed for all-cause mortality [31, 32].

Few epidemiological studies, however, have investigated the impact of clinically diagnosed hypothyroidism on breast cancer prognosis. One study has evaluated the association between clinically diagnosed hypothyroidism, using both diagnoses and prescriptions of levothyroxine, and breast cancer progression [33]. In a Danish population-based breast cancer cohort, the authors observed no association between hypothyroidism, regardless of whether it was diagnosed before or after breast cancer and risk of breast cancer recurrence or all-cause mortality [33]. In contrast, in a Canadian population-based study, a small, significant reduced risk of all-cause mortality was observed in a subset of older breast cancer patients who were levothyroxine users compared to non-users [34]. In another study containing 576 hormone receptor-positive breast cancer patients from a single-centre, levothyroxine users had a significantly shorter disease-free and disease-specific survival compared to non-users [14]. To date, no population-based study has investigated clinical hypothyroidism and risk of breast cancer-specific mortality.

Further investigation into the long-term impact of hypothyroidism on breast cancer outcomes is necessary given the increasing number of breast cancer survivors [2] and the increasing prevalence of hypothyroidism and levothyroxine use [35]. Thus, we aimed to investigate the association between hypothyroidism diagnosed before breast cancer, and separately, hypothyroidism diagnosed after breast cancer, and risk of breast cancer-specific and all-cause mortality in a large contemporary UK population-based cohort.

## Methods

### Data sources

This study utilised linkages between national datasets from Scotland including the Scottish Cancer Registry (SMR06), the Prescribing Information System (PIS), the General/Acute Inpatient and Day Case dataset (SMR01), the Outpatient Attendance dataset (SMR00) and the National Records of Scotland Death Records. The Scottish Cancer Registry captures information on all cancers in Scotland and information on medications was obtained from the PIS which covers all medicines dispensed in the community. The General/Acute Inpatient and Day Case dataset captures hospital diagnoses, and the Outpatient Attendance dataset captures diagnoses and procedures from outpatient clinics. The National Records of Scotland Death Records provided mortality data including date and underlying cause of death. All databases covered the periods from January 1999 to May 2019 except for the PIS which was available from January 2009 to May 2019 and the Cancer Registry which was available until 31st of December 2017. The Community Health Index number (unique to each resident in Scotland) was used to link the individual data sources [36]. The study was approved the Privacy Advisory Committee of the National Health Service (NHS) National Services Scotland (Number: 1617-0374).

### Study design

We identified women with newly diagnosed breast cancer using the International Classification of Diseases 10th revision (ICD10), code C50, between January 2010 and December 2017 from the Scottish Cancer Registry. Women were excluded if they had a previous cancer diagnosis (apart from non-melanoma skin cancer), hyperthyroidism (described later) before breast cancer or less than one year of follow-up. The main outcome of interest was breast cancer-specific mortality, as the primary underlying cause of death, and secondly, all-cause mortality, identified using the National Records of Scotland Death Records.

A separate cohort of female cancer-free controls was also identified and each control was randomly selected, without replacement, using the Community Health Index database, and matched to each breast cancer patient on year of birth. The index date for controls was defined as the date of breast cancer diagnosis in their matched case. Similarly, controls were excluded if they had a history of hyperthyroidism or less than one year of follow-up.

### Thyroid conditions

We identified patients with a diagnosis for hypothyroidism in SMR01 or SMR00 using the ICD10 codes E03.2–E03.9 and E89.0, or at least two dispensed prescriptions for levothyroxine from the PIS [33]. Hypothyroidism before breast cancer was determined as described in the year prior to breast cancer, and hypothyroidism after breast cancer was determined from any point after breast cancer diagnosis. The ICD10 codes E05–E05.9 and E05.0B or at least two dispensed prescriptions for anti-thyroxine medications including, carbimazole and propylthiouracil were used to identify patients with hyperthyroidism in the same time periods [33].

### Covariates

Data available from the Scottish Cancer Registry included age and year of breast cancer diagnosis, TNM cancer stage [37] (based upon pathological stage where recorded, or clinical stage), histological tumour grade, cancer treatments (including surgery, chemotherapy, radiotherapy and endocrine therapy) received within six months of diagnosis, human epidermal growth factor receptor 2 (HER2) status, progesterone receptor (PR) status and ER status. Comorbidities from the Charlson Comorbidity Index (specifically myocardial infarction, congestive heart failure, cerebrovascular accident, peripheral vascular disease, dementia, pulmonary disease, peptic ulcer, liver disease, diabetes, diabetes complications, paraplegia, connective tissue disorder, renal disease and severe liver disease) prior to breast cancer diagnosis were identified from hospital admissions data (SMR00 and SMR01) using previously described ICD10 codes [38]. Aspirin, statin (after diagnosis) and hormone replacement therapy (HRT) (in the year prior to diagnosis) use were identified from the PIS. Deprivation level was determined from the postcode of residence using the 2009 Scottish Index of Multiple Deprivation which uses seven super output area level indices [39].

### Statistical analysis

Cox proportional hazards regression was used to calculate the hazard ratio (HR) and 95% confidence intervals (CIs) for associations between hypothyroidism, diagnosed before and after breast cancer diagnosis, and risk of breast cancer-specific mortality.

In the analysis of hypothyroidism diagnosed before breast cancer, follow-up began at breast cancer diagnosis and ended at one of the following: death, leaving Scotland, hyperthyroidism diagnosis or the end of study period (31st May 2019), as shown in Online Resource 1. The adjusted model contained age at diagnosis, year of diagnosis, deprivation, comorbidities, cancer treatments (treated as time-varying), aspirin and statin use after breast cancer diagnosis, treated as time-varying and lagged by one year from the first dispensed prescription, cancer stage and histological tumour grade.

In analysis of hypothyroidism diagnosed after breast cancer, patients with existing hypothyroidism and those with less than one year of follow-up were excluded. Hypothyroidism was treated as time-varying and was lagged by one year to minimise potential reverse causation. Patients were considered unexposed to hypothyroidism until one year after diagnosis or their second levothyroxine prescription (which ever occurred first) at which point they were considered to be exposed to hypothyroidism for the remainder of follow-up (Online Resource 2). Patients were followed from one year after breast cancer diagnosis until death, date of leaving Scotland, development of hyperthyroidism or the end of the study period. The covariates in the adjusted model were the same as previously stated except cancer treatments were not treated at time-varying as follow-up began after their receipt.

A number of sub-group analyses were conducted including stratification by age (as a proxy for menopausal status: ≤ 55 and > 55 years old [40], ER status (positive or negative), breast cancer diagnosis period (2010–2013 and 2014–2017) and cancer stage (I-III). Additionally, analyses were stratified by cancer treatments received (separately for surgery, chemotherapy, radiotherapy and endocrine therapy) for hypothyroidism diagnosed after breast cancer.

A number of sensitivity analyses were also conducted; firstly, we restricted analysis to patients who were treated for hypothyroidism (received at least two levothyroxine prescriptions). Second, breast cancer from any cause of death on the death certificate was used to define breast cancer mortality. Thirdly, we additionally adjusted for HRT use in the year before cancer diagnosis as it may impact levothyroxine effectiveness [41]. Fourth, a competing risks analysis was conducted to estimate the marginal probability of death from breast cancer in the presence of competing causes of death (any other cause) using the Fine-Gray competing risk model [42]. Fifth, multiple imputation was used to impute missing values of deprivation, cancer stage and tumour grade with chained equations [43]. Briefly, this is a simulation-based approach for managing and analysing missing data under certain assumptions about the randomness of the missing data [44]. The imputation used ordered logit models with deprivation, cancer stage and tumour grade and included the exposure, outcome, cumulative hazard [45], age at diagnosis, year of diagnosis, comorbidities and cancer treatments. In each analysis 20 datasets were imputed and estimates combined using Rubin’s rules [44]. Additionally, for the analysis of hypothyroidism diagnosed before breast cancer, five additional sensitivity analyses were performed; hypothyroidism was modified to include diagnoses and prescriptions for levothyroxine identified within two and three years prior to breast cancer diagnosis for patients with available records, diagnoses of hypothyroidism from the SMR00 and SMR01 were identified at any time before cancer diagnosis and we additionally included incident hypothyroidism after breast cancer diagnosis as a censoring variable, plus we did not adjust for cancer stage, histological tumour grade and cancer treatments received as they may be on the causal pathway in the analysis of hypothyroidism diagnosed before breast cancer. Lastly, in the analysis of hypothyroidism diagnosed after breast cancer, the lag period between a diagnosis and becoming exposed was increased to 2 years. All analyses were repeated for all-cause mortality.

In additional analyses, incidence rates (IRs) for hypothyroidism and corresponding 95% CIs were calculated comparing breast cancer patients and age-matched cancer-free controls without a prior diagnosis of hypothyroidism. Follow-up began at breast cancer diagnosis or index date lagged by one year and ended at the date of hypothyroidism diagnosis, death, leaving Scotland, hyperthyroidism diagnosis or the end of study period (31^st^ May 2019). Cox proportional hazards regression was used to compare incident hypothyroidism in breast cancer patients to population-based controls adjusting for age, comorbidities (described above) and deprivation level.

## Results

**Fig. 1:**
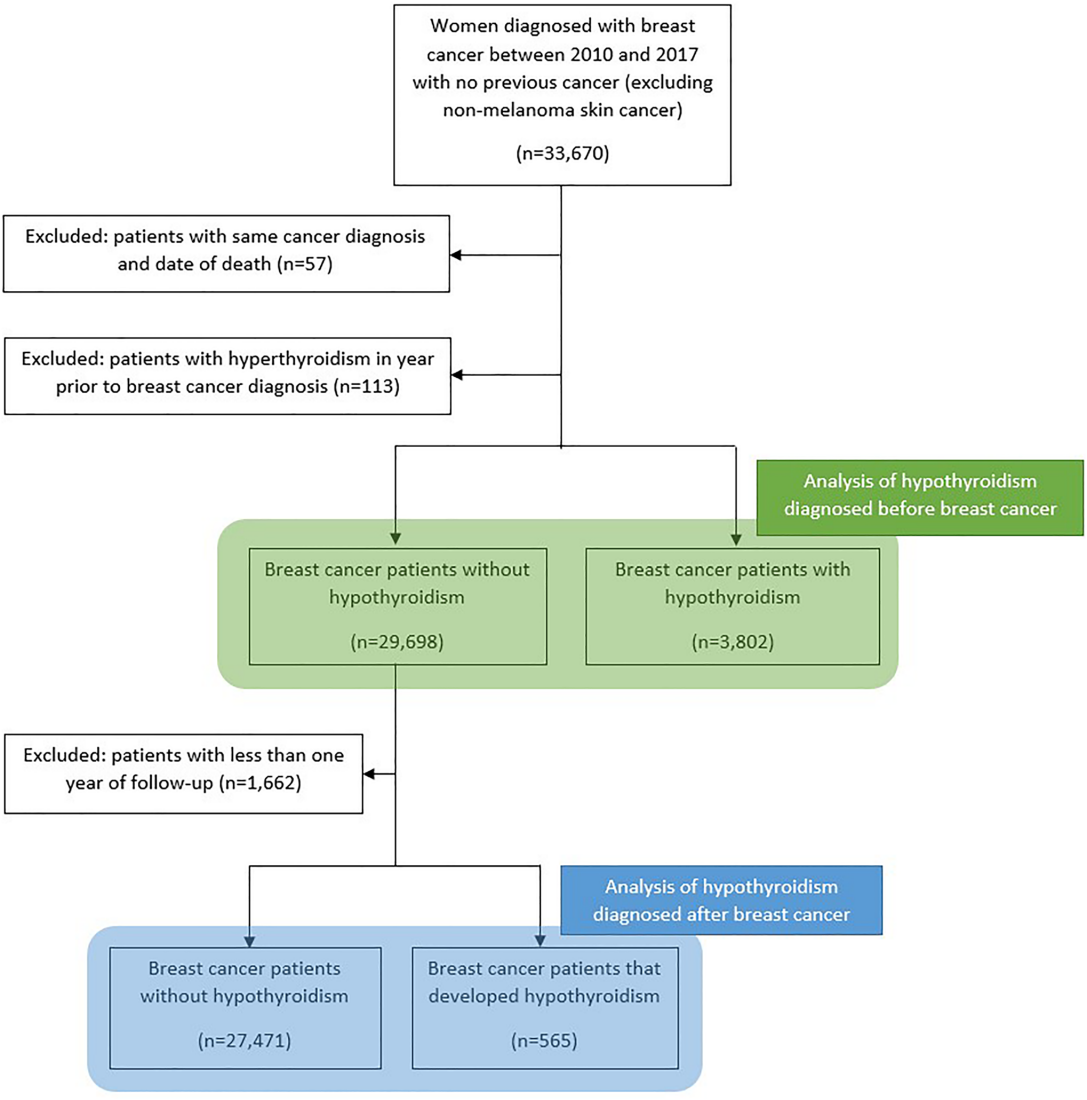
Flow diagram outlining the number of breast cancer patients with hypothyroidism

In total, there were 33,500 breast cancer patients diagnosed between 2010 and 2017 that met the study inclusion criteria, Fig. 1. A total of 3802 breast cancer patients had existing hypothyroidism and of the remaining patients, 565 developed hypothyroidism after breast cancer diagnosis (Fig. 1).

The characteristics of patients with and without hypothyroidism are displayed in Table 1. Breast cancer patients with hypothyroidism before breast cancer were more likely to be older and take medications such as aspirin and statins compared to patients without hypothyroidism. Additionally, they were more likely to receive endocrine therapy and less likely to receive chemotherapy within six months of diagnosis, and less likely to have HER2-positive tumours. A history of comorbidities such as myocardial infarction, congestive heart disease, diabetes, cerebrovascular accident and pulmonary disease was also greater among patients diagnosed with hypothyroidism before breast cancer than patients without.

Patients who developed hypothyroidism after breast cancer were more likely to be diagnosed with breast cancer earlier in the study period and were also more likely to undergo surgery and receive chemotherapy and use HRT in comparison to those who did not develop hypothyroidism (Table 1)

**Table 1:** Characteristics of breast cancer patients with and without hypothyroidism

	Hypothyroidism diagnosed before breast cancer (count (%))		Hypothyroidism diagnosed after breast cancer (count (%))	
	No (n = 29,698)	Yes (n = 3,802)	No (n = 27,471)	Yes (n = 565)
Year of diagnosis				
2010–2011	7383(24.9%)	921(24.2%)	6714(24.4%)	249(44.1%)
2012–2013	7528(25.3%)	968(25.5%)	6939(25.3%)	159(28.1%)
2014–2015	7442(25.1%)	915(24.1%)	6920(25.2%)	116(20.5%)
2016–2017	7345(24.7%)	998(26.2%)	6898(25.1%)	41(7.3%)
Age at diagnosis				
< 50	5414(18.2%)	239(6.3%)	5185(18.9%)	128(22.7%)
50–59	7358(24.8%)	667(17.5%)	7063(25.7%)	163(28.8%)
60–69	7745(26.1%)	1104(29.0%)	7369(26.8%)	141(25.0%)
70–79	5120(17.2%)	922(24.3%)	4673(17.0%)	80(14.2%)
80–89	3237(10.9%)	702(18.5%)	2649(9.6%)	44(< 10%)
≥ 90	824(2.8%)	168(4.4%)	532(1.9%)	< 10
Cancer stage				
1	11,817(39.8%)	1487(39.1%)	11,471(41.8%)	219(38.8%)
2	11,026(37.1%)	1377(36.2%)	10,486(38.2%)	228(40.4%)
3	2401(8.1%)	288(7.6%)	2181(7.9%)	51(9.0%)
4	1780(6.0%)	233(6.1%)	1167(4.2%)	14(2.5%)
Missing	2674(9.0%)	417(11.0%)	2166(7.9%)	53(9.4%)
Tumour grade				
1	3420(11.5%)	406(10.7%)	3284(12.0%)	85(15.0%)
2	13,262(44.7%)	1713(45.1%)	12,647(46.0%)	248(43.9%)
3	10,013(33.7%)	1245(32.7%)	9364(34.1%)	198(35.0%)
Missing	3003(10.1%)	438(11.5%)	2176(7.9%)	34(6.0%)
Deprivation level				
1	4968(16.7%)	661(17.4%)	4527(16.5%)	87(15.4%)
2	5666(19.1%)	764(20.1%)	5168(18.8%)	104(18.4%)
3	6018(20.3%)	807(21.2%)	5578(20.3%)	128(22.7%)
4	6428(21.6%)	807(21.2%)	5981(21.8%)	120(21.2%)
5	6524(22.0%)	753(19.8%)	6127(22.3%)	126(22.3%)
Missing	94(0.3%)	10(0.3%)	90(0.3%)	0(0.0%)
ER status				
Negative	4483(15.1%)	581(15.3%)	3997(14.5%)	89(15.8%)
Positive	24,424(82.2%)	3111(81.8%)	23,039(83.9%)	465(82.3%)
Missing	791(2.7%)	110(2.9%)	435(1.6%)	11(1.9%)
PR status				
Negative	6849(23.1%)	912(24.0%)	6201(22.6%)	122(21.6%)
Positive	16,325(55.0%)	2076(54.6%)	15,469(56.3%)	286(50.6%)
Missing	6524(22.0%)	814(21.4%)	5801(21.1%)	157(27.8%)
HER2 status				
Negative	23,458(79.0%)	3046(80.1%)	22,059(80.3%)	446(78.9%)
Positive	4194(14.1%)	468(12.3%)	3886(14.1%)	82(14.5%)
Missing	2046(6.9%)	288(7.6%)	1526(5.6%)	37(6.5%)
Treatments within 6 months of breast cancer diagnosis				
Surgery	22,561(76.0%)	2755(72.5%)	21,799(79.4%)	475(84.1%)
Chemotherapy	10,125(34.1%)	904(23.8%)	9625(35.0%)	239(42.3%)
Radiotherapy	11,159(37.6%)	1499(39.4%)	10,691(38.9%)	203(35.9%)
Endocrine therapy	18,982(63.9%)	2665(70.1%)	17,925(65.3%)	349(61.8%)
Comorbidities prior to breast cancer diagnosis				
Myocardial infarction	411(1.4%)	81(2.1%)	334(1.2%)	< 10
Congestive heart disease	1171(3.9%)	278(7.3%)	1006(3.7%)	16(2.8%)
Dementia	191(0.6%)	30(0.8%)	123(0.4%)	< 10
Renal failure	110(0.4%)	27(0.7%)	87(0.3%)	< 10
Diabetes	1094(3.7%)	292(7.7%)	910(3.3%)	23(4.1%)
Diabetes complications	57(0.2%)	24(0.6%)	46(0.2%)	< 10
Liver disease	67(0.2%)	19(0.5%)	58(0.2%)	< 10
Paraplegia	93(0.3%)	19(0.5%)	73(0.3%)	< 10
Severe liver disease	39(0.1%)	< 10	30(0.1%)	< 10
Peptic ulcer	398(1.3%)	61(1.6%)	352(1.3%)	< 10
Connective tissue disorder	343(1.2%)	67(1.8%)	299(1.1%)	< 10
Pulmonary disease	1770(6.0%)	319(8.4%)	1554(5.7%)	27(4.8%)
Cerebrovascular accident	922(3.1%)	185(4.9%)	741(2.7%)	< 10
Medication use in year prior to breast cancer diagnosis				
HRT	2725(9.2%)	356(9.4%)	2590(9.4%)	78(13.8%)
Medication use after breast cancer diagnosis				
Aspirin	4551(15.3%)	924(24.3%)	4141(15.1%)	82(14.5%)
Statins	8309(28.0%)	1572(41.3%)	7754(28.2%)	175(31.0%)

*ER*  oestrogen receptor, *PR* progesterone receptor, *HER2* Human epidermal growth factor 2 receptor, *HRT* hormone replacement therapy

### Hypothyroidism diagnosed before breast cancer

In total, 4,210 breast cancer-specific deaths among 33,500 breast cancer patients occurred during follow-up, (Table 2). In the main analysis, unadjusted HRs suggested a significant 19% increased risk of cancer-specific mortality for patients with existing hypothyroidism compared to patients without (HR 1.19, 95% CI 1.09–1.31) but this was attenuated in adjusted analyses (HR 0.99, 95% CI 0.88–1.12), Table 2.

**Table 2:** The risk of cancer-specific and all-cause mortality in breast cancer patients with hypothyroidism compared to without hypothyroidism

			Cancer-specific mortality			All-cause mortality		
	Breast cancer patients (count (%))	Person years	Deaths (count (%))	Crude HR (95% CIs)	Adjusted HR (95% CIs) ^a^	Deaths (count (%))	Crude HR (95% CIs)	Adjusted HR (95% CIs) ^a^
No hypothyroidism	29,698 (88.6)	137,511.2	3,677 (87.3)	Ref	Ref	6,385 (85.8)	Ref	Ref
Hypothyroidism before breast cancer	3,802 (11.4)	16,453.7	533 (12.7)	1.19 (1.09–1.31)	0.99 (0.88–1.12)	1,052 (14.2)	1.37 (1.28–1.46)	1.01 (0.93–1.10)
No hypothyroidism	27,471 (98.0)	107,160.3	2,586 (98.4)	ref	ref	4,680 (98.4)	ref	ref
Hypothyroidism after breast cancer	565 (2.0)	1610.8	42 (1.6)	1.33 (0.98–1.81)	0.97 (0.63–1.49)	78 (1.6)	1.20 (0.96–1.51)	1.07 (0.80–1.43)

^a^ Adjusted for age, year of diagnosis, deprivation, comorbidities prior to breast cancer, aspirin and statin use after breast cancer (lagged by 1 year and treated as time-varying), cancer treatments within 6 month of breast cancer diagnosis (surgery, chemotherapy, radiotherapy and hormone therapy treated as time-varying in before analysis), cancer stage and tumour grade

**Fig. 2:**
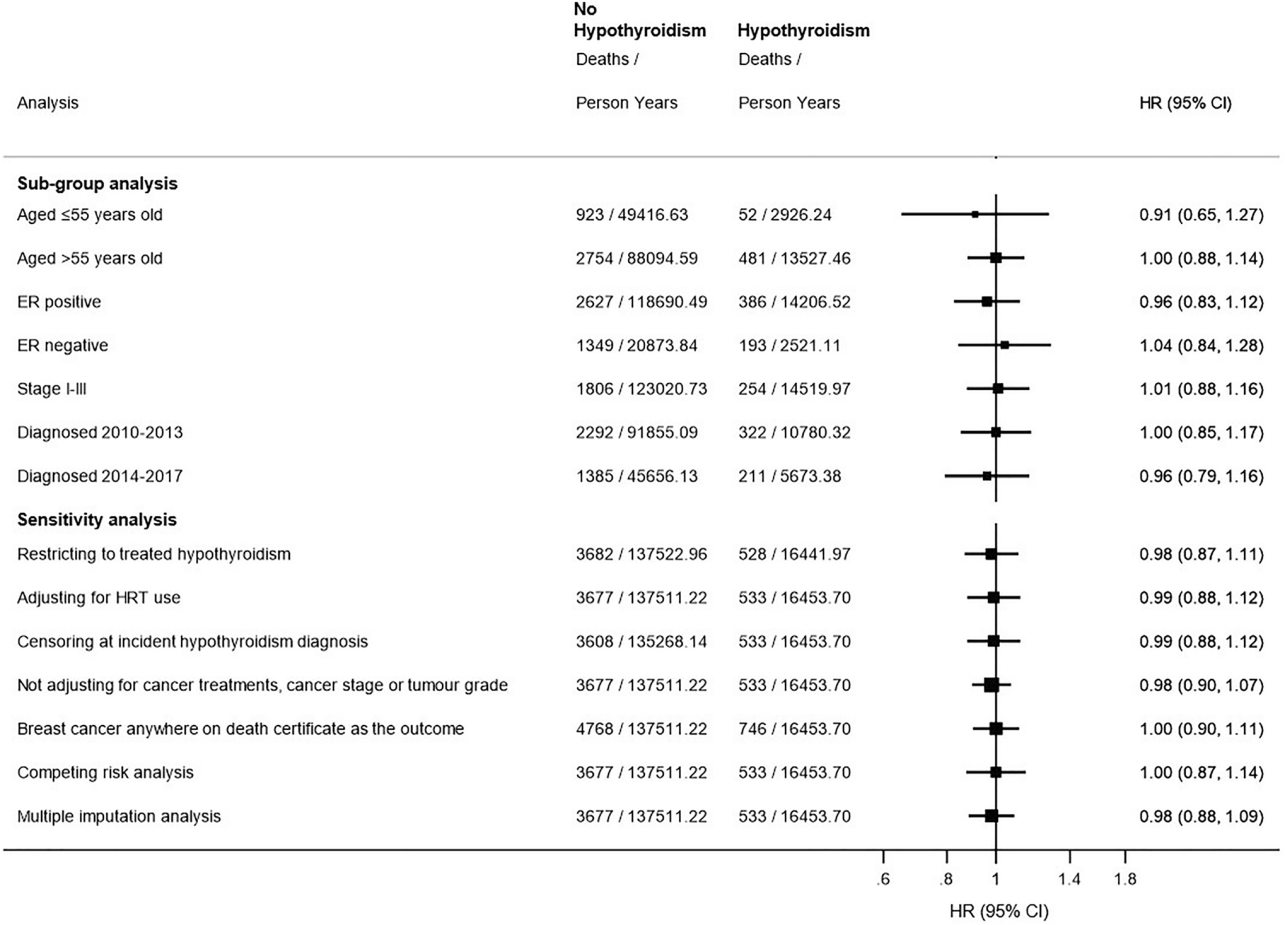
Sub-group and sensitivity analyses for the risk of cancer-specific mortality in patients with hypothyroidism diagnosed before breast cancer compared to patients without hypothyroidism. *ER* oestrogen receptor, *HRT* hormone replacement therapy

Adjusted results for sub-group analyses restricting to age, ER status, cancer stage I-III, and cancer diagnosis period remained largely similar to the main analysis (Fig. 2).

There was no association observed between hypothyroidism diagnosed before breast cancer and the risk of cancer-specific mortality across a number of sensitivity analyses, see Fig. 2. Increasing the exposure period for hypothyroidism diagnosis before breast cancer did not remarkably change the result (2 years: HR 1.00, 95% CI 0.89–1.13 and 3 years: HR 0.87, 95% CI 0.74–1.01), nor did varying the hypothyroidism definition to include hospital diagnoses at any point before breast cancer (HR 0.99, 95% CI 0.87–1.12).

### Hypothyroidism diagnosed after breast cancer

Among 28,036 patients with at least one year of follow-up and without hypothyroidism at breast cancer diagnosis, there were 2,628 breast cancer-specific deaths. The risk of cancer-specific mortality was increased for patients who went on to develop hypothyroidism in unadjusted analyses (HR 1.33, 95% CI 0.98–1.81), but not after adjustment (HR 0.97, 95% CI 0.63–1.49), Table 2.

**Fig. 3:**
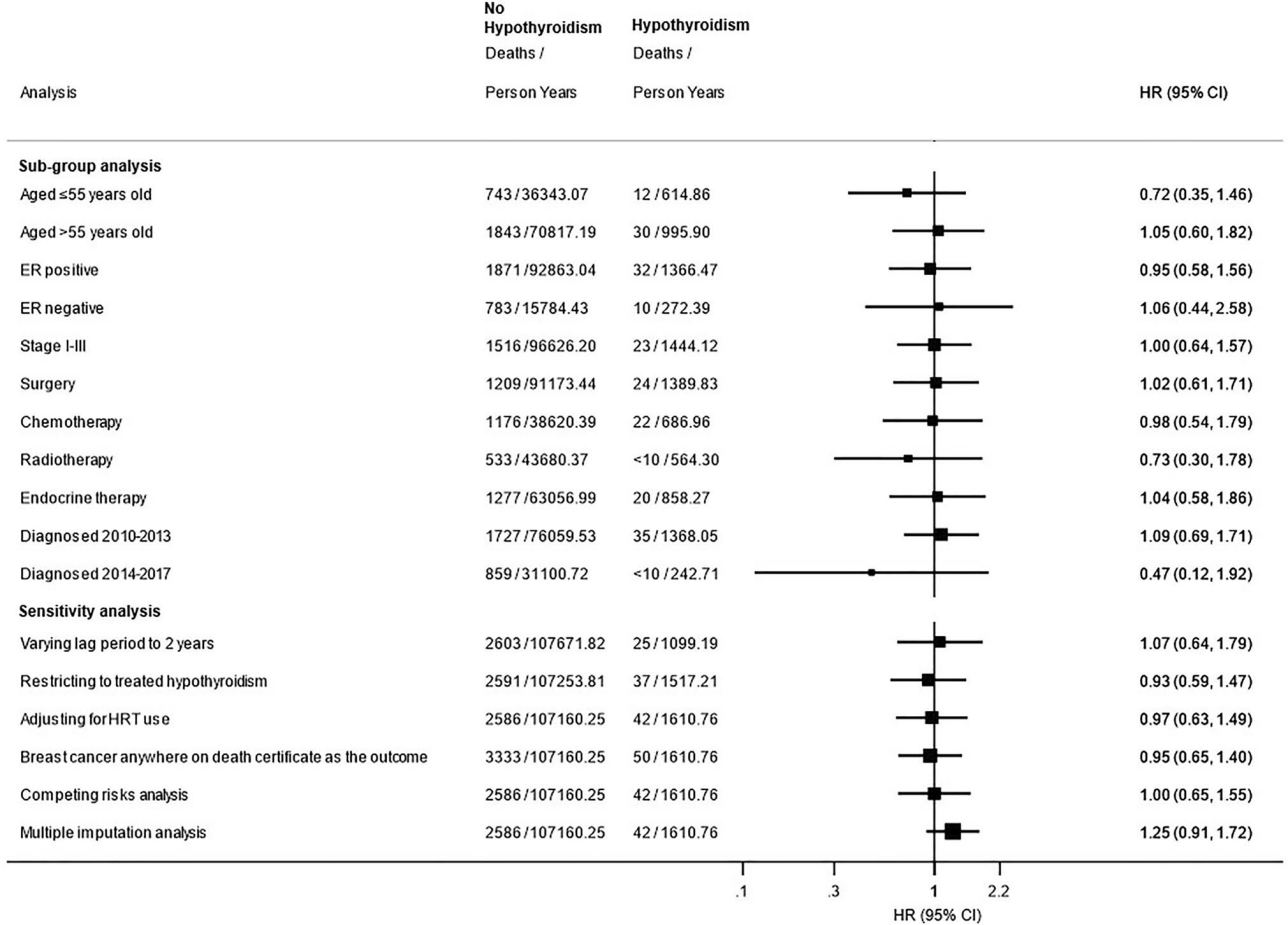
Sub-group and sensitivity analyses for the risk of cancer-specific mortality in patients with hypothyroidism diagnosed after breast cancer compared to patients without hypothyroidism. *ER* oestrogen receptor, *HRT* hormone replacement therapy

In sub-group analyses according to age at breast cancer diagnosis, ER status, cancer stage, cancer treatments received and cancer diagnosis period, adjusted results remained largely similar to the main analysis and no significant associations were observed (Fig. 3).

In sensitivity analyses (Fig. 3), results were similar to the main analysis. Using multiple imputation to impute missing values for deprivation, cancer stage and tumour grade, HRs were raised, although not significantly (HR 1.25, 95% CI 0.91–1.72).

No association was observed for hypothyroidism, diagnosed before or after breast cancer, and all-cause mortality as shown in Table 2 and Online Resources 3 and 4.

### Hypothyroidism in breast cancer patients and matched cancer-free controls

There were a total of 24,203 matched cancer-free controls identified, (Online Resource 5), including 448 incident hypothyroidism diagnoses. The IR for hypothyroidism was higher in breast cancer patients compared to matched controls (5.06, 95% CI 4.65–5.51, and 4.48, 95% CI 4.08–4.91, per 1,000 person years respectively) and corresponded to a 14% higher risk of developing hypothyroidism (adjusted HR 1.14, 95% CI 1.01–1.30), Table 3.

**Table 3:** Hypothyroidism in breast cancer patients compared to age-matched cancer-free controls

	Number (%)	Events (%)	Person years	IR (95% CI)	Crude HR (95% CI)	Adjusted HR (95% CI) ^a^
Cancer-free controls	24,203 (46.5)	448 (45.3)	100,038.42	4.48 (4.08–4.91)	ref	ref
Breast cancer patients	27,888 (53.5)	540 (54.7)	106,604.3	5.06 (4.65–5.51)	1.13 (0.99–1.28)	1.14 (1.01–1.30)

^a^ Adjusted for age, year of diagnosis/year of index, deprivation, comorbidities prior to breast cancer diagnosis/index date

## Discussion

Our results are similar to a recent population-based study conducted in Denmark [33] that reported no association between hypothyroidism, diagnosed before (HR 1.01, 95% CI 0.87–1.19) or after breast cancer diagnosis (HR 0.93, 95% CI 0.75–1.16) and breast cancer recurrence and results were similar for all-cause mortality [33]. We used similar definitions to identify patients with hypothyroidism using both hospital diagnoses and levothyroxine prescription records, and were able to adjust for deprivation level and cancer treatments received, as opposed to cancer treatment intent in the Danish study. Other differences included the exposure period, where we restricted to the year before breast cancer diagnosis to identify patients with hypothyroidism, but results remained similar when we extended the exposure period to two and three years before cancer diagnosis. In contrast, in a Canadian population-based breast cancer cohort [34], a small significant reduced risk of all-cause mortality was observed in a sub-group of patients who were levothyroxine users before breast cancer compared to non-users (HR 0.87, 95% CI 0ER and PR positive tumours.77–0.98). However, as this was a secondary analysis, key covariates such as age at diagnosis, cancer stage, tumour grade and cancer treatments were not considered in the propensity matching process, which may have led to confounding of the estimate [34]. In a single-centre study conducted in the USA, breast cancer patients with ER and PR-positive tumours receiving levothyroxine had a significantly worse disease-free and disease-specific survival than patients not receiving levothyroxine [14]. However, this study only included breast cancer patients who were stage I and lymph node negative, comprised a small number of patients with who received  levothyroxine (n = 38) and only adjusted for age, tumour size and tumour grade, limiting the interpretation of results.

In additional analyses we demonstrated a higher incidence rate, and risk of hypothyroidism after adjustment for confounders, in breast cancer patients compared to controls, which was similar to other studies [19–24].

Overall, our results are reassuring in that hypothyroidism, diagnosed either before or after breast cancer, was not associated with the risk of death from breast cancer. Further large population-based studies are needed to corroborate our findings as this is the first study to investigate breast cancer mortality specifically, and studies should ideally be conducted in different populations to explore the impact of ethnicity and iodine status, as the Scottish and Danish populations are both predominantly white and iodine sufficient or only mildly deficient [7, 46].

Our study has a number of strengths. Firstly, we analysed a large population-based cohort of breast cancer patients from the Scottish Cancer Registry, which has previously been shown to have a high completeness [47] and also included important clinical covariates such as cancer stage, tumour grade and cancer treatments received. Second, as hypothyroidism may be more commonly diagnosed in primary care settings, we identified hypothyroidism using prescription records for levothyroxine from the PIS, which covers all dispensed prescriptions in Scotland. Levothyroxine is not available over-the-counter in the UK and so it is likely that all levothyroxine prescriptions during the study period were captured. Third, in analysis of hypothyroidism diagnosed after breast cancer, a time-varying approach was used to avoid introducing immortal time bias and a lag was applied to avoid reverse causation [48, 49]. Furthermore, we also had access to a matched cancer-free control cohort which allowed us to compare the incidence of hypothyroidism to breast cancer patients.

However, some limitations should be considered. We did not have information on blood thyroid hormone levels and so could not investigate untreated subclinical hypothyroidism or undiagnosed hypothyroidism. We therefore cannot exclude potential misclassification of hypothyroidism, which may have biased our findings towards the null. In the UK, however, there has been a substantial increase in the rates of treated subclinical hypothyroidism with levothyroxine in the past two decades, doubling from 1996 to 2006 [50], which may have lowered the proportion of untreated subclinical hypothyroidism in the study population. Moreover, in analysis of hypothyroidism after diagnosis, breast cancer patients may be more likely to have increased interaction with healthcare professionals during their diagnosis and follow-up [51] compared to the general female population and therefore may be more likely to have comorbidities [52] including hypothyroidism, recognised and treated, for which levothyroxine is standard [53]. As anticipated, the majority of hypothyroidism cases were identified by levothyroxine prescriptions alone (76.4%) similar to other investigations of thyroid disorders in population-based cohorts that utilised drug prescription and hospital records [33, 54]. However, given the prevalent use of levothyroxine (i.e. T4 substitution), any impact of hypothyroidism (i.e. low T4 levels) on breast cancer progression may have been diluted in our analysis. Nonetheless, this is reflective of clinical practice making our results clinically relevant, and there is evidence that levothyroxine treatment may not completely resolve hypothyroid T4 levels in peripheral and tumour tissue, even when a patient has serum TSH levels within an acceptable range [55, 56]. There is also the potential for residual confounding by lifestyle factors such as body mass index, smoking and alcohol intake, which we did not have information on. However, the evidence for the impact of lifestyle exposures after breast cancer diagnosis and risk of cancer-specific mortality is less consistent [57]. As we lacked detailed information on the type of chemotherapy and radiotherapy received there may be residual confounding by these factors and by cancer stage and tumour grade for which there was some missing data. However, in sensitivity analyses using multiple imputation to impute missing values for stage and grade we observed a similar result that overlapped with the main analysis. Lastly, although some misclassification of breast cancer deaths may have occurred, methodological studies have suggested that in comparative studies where differential misclassification of death is unlikely, as in our study, effect estimates are unlikely to be affected [58].

## Conclusion

In this large UK population-based study, there was no association observed between hypothyroidism, diagnosed either before or after breast cancer and breast cancer-specific or all-cause mortality. Given the high prevalence of hypothyroidism in older women, these findings provide some reassurance to breast cancer patients and their treating physicians; however, further study in other populations is required to verify our findings.

## Supplementary Information

Electronic supplementary material 1 (JPG 27.2 kb)

Electronic supplementary material 2 (JPG 63.8 kb)

Electronic supplementary material 3 (JPG 126 kb)

Electronic supplementary material 4 (JPG 127 kb)

Electronic supplementary material 5 (DOCX 40.7 kb)

## Data Availability

Data were obtained and analysed, within a virtual safe haven, under strict licence conditions from the National Health Service National Services Scotland which do not permit data sharing. However, a researcher would be able to reconstruct these datasets and replicate these analyses after obtaining similar approvals from the National Health Services National Services Scotland.
