# Supplementary figures and images for: Cancer-specific mortality in breast cancer patients with hypothyroidism: a UK population-based study
Source: Breast Cancer Res Treat. 2022 Jul 31;195(2):209–21. doi: 10.1007/s10549-022-06674-5 (PMC9374643; doi:10.1007/s10549-022-06674-5)

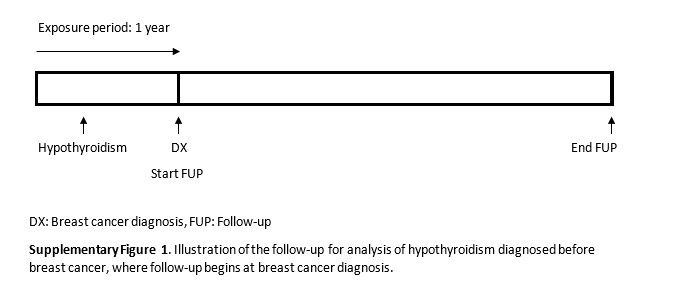

Supplement: Supplementary file 1 — Electronic supplementary material 1 (JPG 27.2 kb) [file 10549_2022_6674_MOESM1_ESM.jpg]

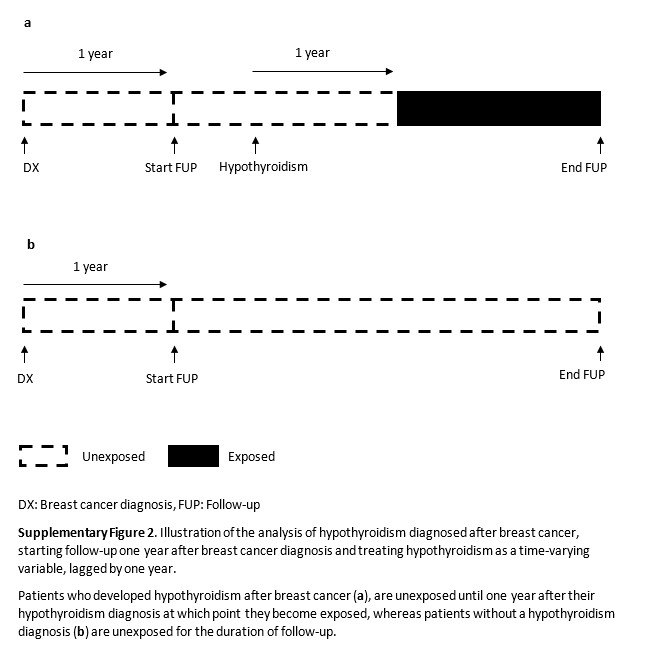

Supplement: Supplementary file 2 — Electronic supplementary material 2 (JPG 63.8 kb) [file 10549_2022_6674_MOESM2_ESM.jpg]

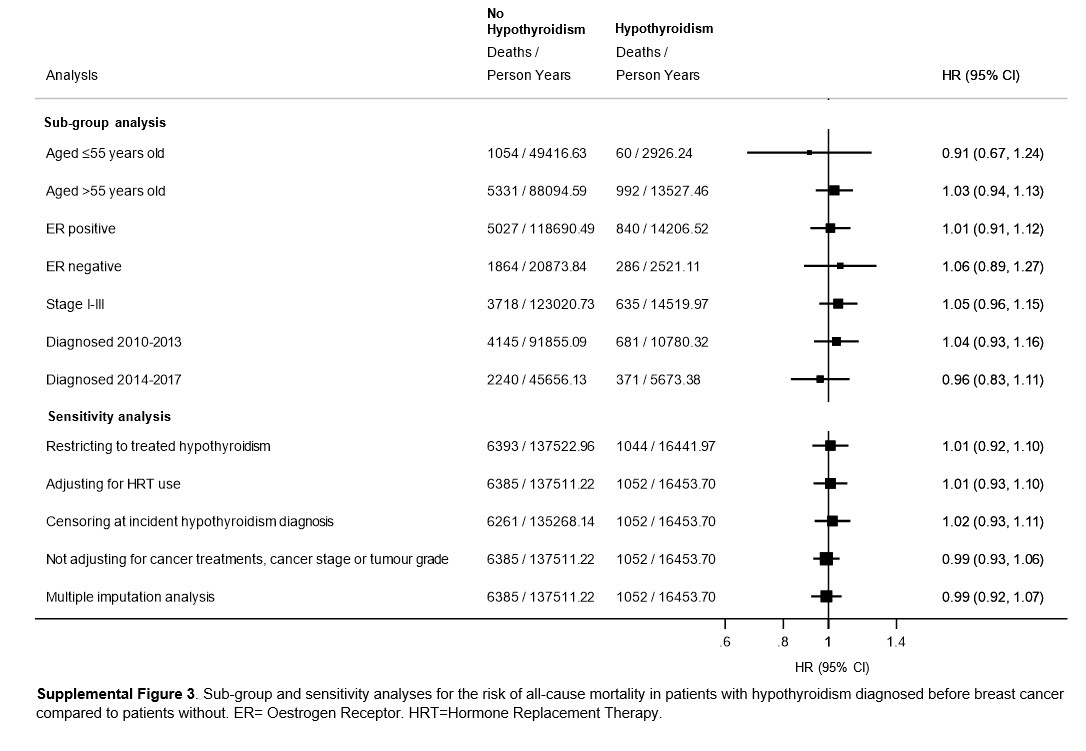

Supplement: Supplementary file 3 — Electronic supplementary material 3 (JPG 126 kb) [file 10549_2022_6674_MOESM3_ESM.jpg]

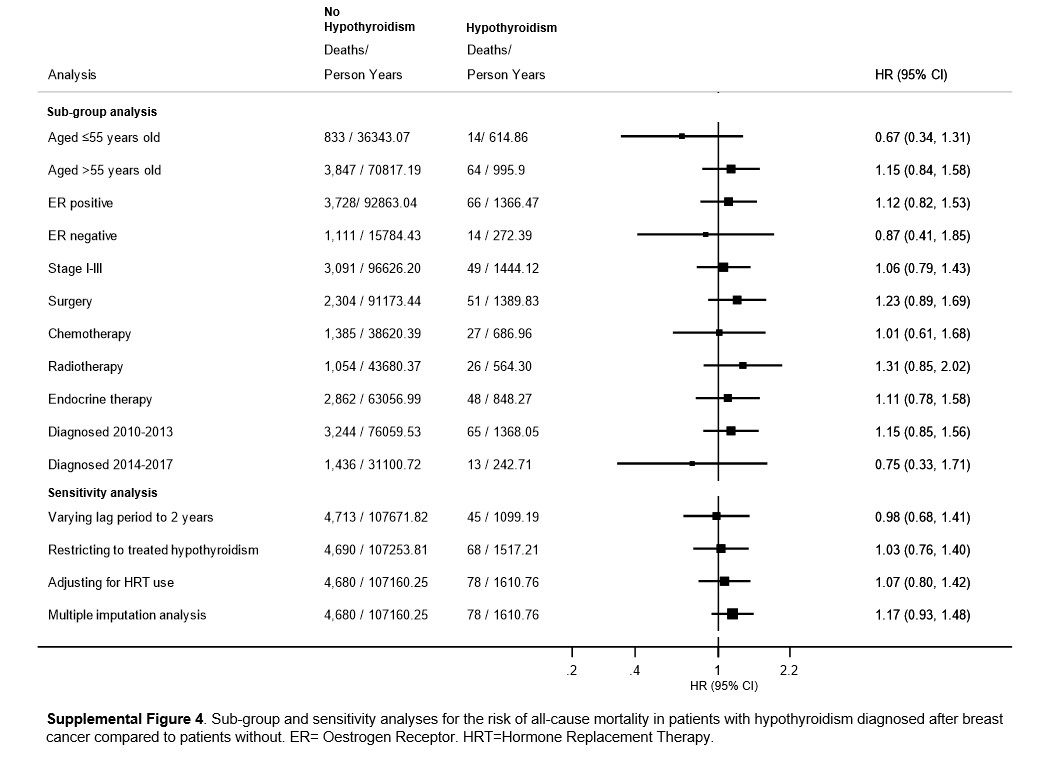

Supplement: Supplementary file 4 — Electronic supplementary material 4 (JPG 127 kb) [file 10549_2022_6674_MOESM4_ESM.jpg]
