# Supplementary material for: Cancer-specific mortality in breast cancer patients with hypothyroidism: a UK population-based study
Source: Breast Cancer Res Treat. 2022 Jul 31;195(2):209–21. doi: 10.1007/s10549-022-06674-5 (PMC9374643; doi:10.1007/s10549-022-06674-5)
Supplement: Supplementary file 5 — Electronic supplementary material 5 (DOCX 40.7 kb) [file 10549_2022_6674_MOESM5_ESM.docx]

Women diagnosed with breast cancer between 2010 and 2017 with no previous cancer (excluding non-melanoma skin cancer) (n=33,670)

Excluded: patients with hyperthyroidism in year prior to breast cancer diagnosis (n=113)

Breast cancer patients without hypothyroidism at diagnosis (n=28,036)

Excluded: patients with less than one year of follow-up (n=1,662)

Breast cancer patients without hypothyroidism (n=29,698)

Breast cancer patients with hypothyroidism (n=3,802)

Breast cancer patients (n=27,888) *

Excluded: patients who developed hypothyroidism within one year of breast cancer diagnosis (n=148)

Excluded: patients whose date of cancer diagnosis was the same as death (n=57)

Controls with hypothyroidism (n=3,878)

Controls without hypothyroidism at diagnosis (n=29,099)

Cancer-free controls with index date between 2010 and 2017 (n=33,669)

Excluded: controls with hyperthyroidism in year prior to index date (n=107)

Excluded: controls whose index date was the same as date of death (n=57)

Controls without hypothyroidism (n=29,684)

Excluded: controls with less than one year of follow-up (n=585)

Excluded: controls who developed hypothyroidism within one year of index date (n=142)

Controls (n=28,957)

Controls (n=24,203)

Excluded: unmatched controls (n=4,754)

**Supplementary Fig 5.** Flow diagram for patients and controls included in analysis of incident hypothyroidism

*The total number of breast cancer patients differs to the main analysis as hypothyroidism was not lagged
